# Draft Genome Sequence of Salmonella bongori N19-781, a Clinical Strain from a Patient with Diarrhea

**DOI:** 10.1128/MRA.00691-19

**Published:** 2019-07-18

**Authors:** Marc J. A. Stevens, Nicole Cernela, Andrea Müller, Roger Stephan, Guido Bloemberg

**Affiliations:** a Institute for Food Safety and Hygiene, Vetsuisse Faculty, University of Zurich, Zurich, Switzerland; b Swiss National Center for Enteropathogenic Bacteria and Listeria (NENT), University of Zurich, Zurich, Switzerland; Georgia Institute of Technology

## Abstract

The genome of Salmonella bongori N19-781, a strain isolated from a patient with diarrhea, was sequenced. It consists of a 4.5-Mbp chromosome.

## ANNOUNCEMENT

*Salmonella* strain N19-781 was isolated from a stool sample (using selenite enrichment and cultivation on Hektoen agar and Elite ChromAgar; bioMérieux, Marcy-l’Étoile, France) of a 10-month-old symptomatic female patient residing in the canton of Geneva (Switzerland). According to the White-Kauffmann-Le Minor scheme (1), the strain was serotyped as Salmonella bongori 48:z_35_:−. S. bongori is mostly associated with cold-blooded animals (2). Human infections with S. bongori have hardly been reported, although persistent endemicity of S. bongori, with multiple human cases, has been described in southern Italy (3).

The strain N19-781 was grown overnight (o/n) on sheep blood agar at 37°C prior to genomic DNA isolation using a DNA blood and tissue kit (Qiagen, Hombrechtikon, Switzerland). The DNA was prepared using a Nextera DNA Flex sample preparation kit (Illumina, San Diego, CA, USA), which produces transposome-based libraries that were sequenced on a MiniSeq sequencer (Illumina).

The sequencing output was 1,014,820 150-bp paired-end reads. Reads were checked for quality using the software package FastQC 0.11.7 (Babraham Bioinformatics, Cambridge, UK). Both Illumina read files passed the standard quality checks of FastQC, with the exception of the module “Per Base Sequence Content,” which returned a failure. Such failure is common for transposome-based libraries and was therefore ignored (https://www.bioinformatics.babraham.ac.uk/projects/fastqc/Help/3%20Analysis%20Modules/). Reads were assembled using the SPAdes 3.0-based software Shovill 1.0.4 (4, 5) using default settings. The assembly was filtered, retaining contigs of >500 bp. The draft genome of S. bongori N19-781 consists of 4,519,152 bp divided over 50 contigs, with an *N*_50_ length of 314.9 kbp and a largest contig size of 602,874 kbp. The GC content of the genome is 51.24 mol%. The genome harbors 4,351 genes, including 73 tRNAs, as predicted by the NCBI Prokaryotic Genome Annotation Pipeline (6). The classification of strain N19-781 as S. bongori was confirmed by KmerFinder 3.1 (7) using the genome sequence as input and by >98.9% average nucleotide identity to other S. bongori genomes (data not shown).

The genome sequence of this clinical Salmonella bongori strain might provide information about virulence factors in this species. It is one of the few available genomes of clinical strains of this *Salmonella* species.

### Data availability.

This whole-genome shotgun project has been deposited at DDBJ/ENA/GenBank under the accession number VCNN00000000. The version described in this paper is the first version, VCNN01000000. Reads were deposited in the Sequence Read Archive (SRA) under study number PRJNA545245, experiment number SRX5937612, and run number SRR9164837.
